# Breast cancer risk assessment for prescription of menopausal hormone therapy in women with a family history of breast cancer: an epidemiological modelling study

**DOI:** 10.3399/BJGP.2023.0327

**Published:** 2024-07-09

**Authors:** Catherine Huntley, Bethany Torr, Grace Kavanaugh, Angela George, Helen Hanson, Katie Snape, John Broggio, Louise Glasgow, Marc Tischkowitz, D Gareth Evans, Antonis C Antoniou, Clare Turnbull

**Affiliations:** Division of Genetics and Epidemiology, Institute of Cancer Research, London; National Cancer Registration and Analysis Service, National Disease Registration Service, NHS England, London.; Division of Genetics and Epidemiology, Institute of Cancer Research, London.; Division of Genetics and Epidemiology, Institute of Cancer Research, London.; Division of Genetics and Epidemiology, Institute of Cancer Research, London; Royal Marsden NHS Foundation Trust, London.; Division of Genetics and Epidemiology, Institute of Cancer Research, London; Peninsula Regional Genetics Service, Royal Devon University Healthcare NHS Foundation Trust, Exeter; Department of Clinical and Biomedical Sciences, University of Exeter Medical School, Exeter.; South West Thames Regional Genetics Service, St George’s University Hospitals NHS Foundation Trust, London; St George’s University of London, London.; National Cancer Registration and Analysis Service, National Disease Registration Service, NHS England, London.; Village Health Group Primary Care Practice, Nottingham.; Department of Medical Genetics, National Institute for Health Research, Cambridge Biomedical Research Centre, University of Cambridge, Cambridge.; Division of Evolution, Infection and Genomics, University of Manchester, Manchester; Manchester Centre for Genomic Medicine and North West Laboratory Genetics Hub, Manchester University NHS Foundation Trust, Manchester Academic Health Science Centre, Manchester.; Centre for Cancer Genetic Epidemiology, Department of Public Health and Primary Care, University of Cambridge, Cambridge.; Division of Genetics and Epidemiology, Institute of Cancer Research, London; National Cancer Registration and Analysis Service, National Disease Registration Service, NHS England, London; Royal Marsden NHS Foundation Trust, London.

**Keywords:** breast cancer, epidemiology, genetics

## Abstract

**Background:**

Menopausal hormone therapy (MHT) can alleviate menopausal symptoms but has been associated with an increased risk of breast cancer. MHT prescription should be preceded by individualised risk/benefit evaluation; however, data outlining the impact of family history alongside different MHT therapeutic approaches are lacking.

**Aim:**

To quantify the risks associated with MHT use in women with varying breast cancer family histories of developing and dying from breast cancer.

**Design and setting:**

An epidemiological modelling study for women in England using the BOADICEA breast cancer prediction model and data relating to MHT use and breast cancer risk taken from research by the Collaborative Group on Hormonal Factors in Breast Cancer.

**Method:**

The risk of developing and dying from breast cancer between the ages of 50 and 80 years was modelled in women with four different breast cancer family history profiles: ‘average’, ‘modest’, ‘intermediate’, and ‘strong’ by using 1) background risks of breast cancer by age and family history, 2) relative risks for breast cancer associated with MHT use, and 3) 10-year breast cancer-specific net mortality rates. This study modelled use of combined oestrogen-progestogen MHT (cyclical or continuous) and oestrogen-only MHT.

**Results:**

For a woman of ‘average’ family history taking no MHT, the cumulative breast cancer risk (age 50–80 years) is 9.8%, and the risk of dying from the breast cancer is 1.7%. In this model, 5 years’ exposure to combined-cyclical MHT (age 50–55 years) was calculated to increase these risks to 11.0% and 1.8%, respectively. For a woman with a ‘strong’ family history taking no MHT, the cumulative breast cancer risk is 19.6% (age 50–80 years), and the risk of dying from the breast cancer is 3.2%. With 5 years’ exposure to MHT (age 50–55 years), this model showed that these risks increase to 22.4% and 3.5%, respectively.

**Conclusion:**

In this model, both family history and MHT are associated with increased risk of breast cancer. Estimates of the risks of breast cancer associated with MHT for women with different family histories can be used to support decision making around MHT prescription for women experiencing menopausal symptoms.

## Introduction

Menopausal hormone therapy (MHT) has been widely prescribed since the 1970s for the management of symptoms associated with female menopause, but has in multiple studies been associated with increased risk of breast cancer, which varies by MHT preparation and duration.^1^^–^3 However, in addition to age and MHT exposure, there are a number of additional risk factors for breast cancer, of which family history is one of the strongest.^4^ Administration of MHT in the context of elevated baseline breast cancer risk is of potential concern to patients and clinicians, but there are limited data available regarding the impact of different patterns of MHT administration on breast cancer risk (or mortality) in the context of differing patterns of family history.^5^^,^^6^

To address this, this study undertook modelling for hypothetical unaffected 50-year-old female consultands of four different profiles of family history: 1) an ‘average’ woman, that is, with family history unknown; 2) a woman with a ‘modest’ family history comprising a single first-degree relative affected with breast cancer at age 60 years; 3) a woman with an ‘intermediate’ family history comprising a single first-degree relative affected with breast cancer at age 40 years; and 4) a woman with a ‘strong’ family history comprising two first-degree relatives affected with breast cancer at age 50 years. (Note that the terms ‘strong’, ‘intermediate’, and ‘modest’ describe family histories constructed for this analysis and do not correspond to the lifetime breast cancer risk definitions used by the National Institute for Health and Care Excellence [NICE] of ‘high-risk’ and ‘moderate-risk’.)

Exposure to four different types of systemic (oral) MHT was considered:

- combined oestrogen-progestogen (combined-all);
  ○ progestogen administered cyclically (intermittently, sequentially), for example, for 10–14 days per month (combined-cyclical);
  ○ progestogen administered continuously (daily, bleed-free) on all days of the month (combined-continuous); or
- oestrogen-only.

**Table:** How this fits in

Prospective longitudinal studies (such as the Collaborative Group on Hormonal Factors in Breast Cancer [CGHFBC]) have enabled the estimation of relative risks of breast cancer associated with different durations of exposure to and formulations of menopausal hormonal therapy (MHT). Risk models such as BOADICEA enable prediction of age-related breast cancer risk according to the extent and pattern of breast cancer family history. This study undertook integration of these two data sources (namely the CGHFBC datasets and the BOADICEA model) in order to model annual and 5-year risks for breast cancer incidence for the age window 50–80 years for hypothetical unaffected female consultands with different patterns of MHT exposure and different patterns of breast cancer family history, also generating predictions for breast cancer-specific death. This study modelled combined and oestrogen-only MHT but lacked data for analyses of newer types of MHT such as micronised progesterone or non-oral preparations.

Data were lacking for newer types of MHT, such as micronised progesterone or non-oral preparations, to include in risk analyses.

Exposure was considered for three different MHT exposure durations (1 year, 5 years, and 10 years), evaluating 1) a woman’s likelihood of developing breast cancer over 5 years, 10 years, and cumulatively up to age 80 years, and 2) her likelihood of dying from a breast cancer diagnosed during this period.

## Method

Baseline risks (without MHT) were estimated using the validated BOADICEA (version 6) breast cancer prediction model, assuming the UK age-specific and calendar period-specific population incidences for invasive breast cancer.^7^^–^9 Estimates for breast cancer relative risks associated with ‘current’ and ‘past’ MHT use were obtained from the Collaborative Group on Hormonal Factors in Breast Cancer (CGHFBC) for four types of MHT: combined-all, combined-cyclical, combined-continuous, and oestrogen-only, where the relative risks for combined-cyclical and combined-continuous MHT were calculated respectively as one-sixth lower and one-sixth higher than those for combined-all MHT, as per CGHFBC findings.^10^

For each MHT preparation, the relative risks from CGHFBC were used relating to each of three durations of MHT administration: age 50.0–51.0 years (1 year), age 50.0–55.0 years (5 years), and age 50.0–60.0 years (10 years) (see Supplementary Table S1 for details). To calculate the absolute risk of breast cancer for each scenario, the annual breast cancer incidence was calculated; the cumulative risk of developing breast cancer between ages 50.0 and age 50.0 *+ x* years in the absence of mortality was then calculated using standard survival analysis theory.

From the CGHFBC meta-analysis of prospective epidemiological studies, MHT was found to increase the relative risk of breast cancer most markedly during the exposed period (‘current use’).^10^ However, breast cancer risk remains elevated for a subsequent ‘legacy period’ following cessation of MHT, with the magnitude relative risk during this time influenced by the duration of MHT exposure (‘past use’).^10^ Thus, in the CGHFBC data the duration of MHT use is thought to have a dual effect: first, accruing risk for longer not only because of ‘current use’ but also influencing the magnitude of relative risk applied during the legacy period (for ‘past use’).

Ten-year breast cancer-specific net mortality rates from 2008–2017 were used stratified by 10-year age-band of diagnosis from the National Disease Registration Service, NHS England.^11^ Mortality rates were considered separately for diagnoses of all invasive breast cancers, and oestrogen receptor (ER)-positive invasive breast cancers. To calculate the baseline breast cancer-specific net mortality associated with the specific family history, the 10-year net (breast cancer-specific) mortality rate was applied for all breast cancers to the per-decade baseline cumulative breast cancer risk (no MHT) for each consultand profile. For additional breast cancer-specific mortality consequent from MHT exposure, the 10-year breast cancer-specific mortality rate for ER-positive breast cancers was applied to the per-decade MHT-related cumulative breast cancer risk, under the assumption that MHT confers risk of ER-positive breast cancer only.^12^ The breast cancer-specific baseline mortality was summed with the MHT-related mortality for each decade 50.0–60.0 years, 60.0–70.0 years, and 70.0–80.0 years, and then in total for breast cancers diagnosed during the age window of 50.0– 80.0 years. (See Supplementary Table S2 for details of additional assumptions applied in the risk modelling.) See Supplementary Methods for additional details about the methods.

## Results

For varying patterns of MHT administration, Table 1 presents the estimated cumulative risk to age 80 years of developing a first breast cancer for four profiles of unaffected 50-year-old female consultands. Table 2 presents the corresponding risks of dying from a breast cancer diagnosed aged 50–80 years. For the ‘average’ 50-year-old woman in the population (with an unknown cancer family history) with no MHT, the cumulative risk of developing breast cancer is 2.7% to age 60 years, 6.2% to age 70 years, and 9.8% to age 80 years, which is respectively increased to 3.5%, 7.5%, and 11.0% with 5 years (age 50–55 years) and to 4.5%, 8.9%, and 12.4% with 10 years (age 50–60 years) of combined-cyclical MHT.

**Table 1a.** Cumulative risk of developing breast cancer from age 50.0–80.0 years, according to family history and MHT use for oestrogen-only and combined-all MHT

**Family** **history**	**MHT type**	**Current age, years**	**51.0**	**52.0**	**53.0**	**54.0**	**55.0**	**60.0**	**65.0**	**70.0**	**75.0**	**80.0**	**Likelihood of** **developing** **breast cancer** **age 50–80.** **One in:**	**Likelihood of** **developing breast** **cancer age 50–80** **attributable to** **MHT. One in:**
**Average woman**		Population risk	**0.3%**	**0.5%**	**0.8%**	**1.0%**	**1.3%**	**2.8%**	**4.4%**	**6.3%**	**8.1%**	**9.9%**	10.1	—

	None	No MHT	**0.2%**	**0.5%**	**0.7%**	**1.0%**	**1.2%**	**2.7%**	**4.3%**	**6.2%**	**8.0%**	**9.8%**	10.2	—

	Oestrogen only	MHT used age 50.0–51.0	0.3%	0.6%	0.8%	1.1%	1.3%	2.9%	4.6%	6.6%	8.4%	10.2%	9.8	256
		MHT used age 50.0–55.0	0.3%	0.6%	0.9%	1.3%	1.5%	3.1%	4.9%	6.9%	8.7%	10.5%	9.5	148
		MHT used age 50.0–60.0	0.3%	0.6%	0.9%	1.3%	1.5%	3.3%	5.1%	7.3%	9.0%	10.8%	9.2	98

	Combined — all types	MHT used age 50.0–51.0	0.3%	0.5%	0.7%	1.1%	1.3%	2.9%	4.6%	6.6%	8.4%	10.2%	9.8	256
		MHT used age 50.0–55.0	0.3%	0.8%	1.1%	1.6%	1.9%	3.7%	5.5%	7.7%	9.5%	11.3%	8.9	67
		MHT used age 50.0–60.0	0.3%	0.8%	1.1%	1.6%	1.9%	4.8%	6.9%	9.4%	11.2%	12.9%	7.7	32

**Modest family history** **(affected FDR age 60)**	None	No MHT	**0.4%**	**0.8%**	**1.2%**	**1.6%**	**2.0%**	**4.3%**	**6.7%**	**9.3%**	**11.7%**	**13.8%**	7.2	—

	Oestrogen only	MHT used age 50.0–51.0	0.5%	0.9%	1.4%	1.8%	2.2%	4.6%	7.2%	9.9%	12.3%	14.4%	6.9	170
		MHT used age 50.0–55.0	0.5%	1.0%	1.5%	2.0%	2.6%	5.0%	7.6%	10.4%	12.7%	14.8%	6.7	98
		MHT used age 50.0–60.0	0.5%	1.0%	1.5%	2.0%	2.6%	5.3%	8.0%	10.9%	13.2%	15.3%	6.5	66

	Combined — all types	MHT used age 50.0–51.0	0.5%	0.8%	1.3%	1.7%	2.1%	4.6%	7.1%	9.8%	12.3%	14.4%	6.9	170
		MHT used age 50.0–55.0	0.6%	1.3%	1.9%	2.5%	3.2%	5.9%	8.6%	11.6%	14.0%	16.0%	6.2	45
		MHT used age 50.0–60.0	0.6%	1.3%	1.9%	2.5%	3.2%	7.6%	10.7%	14.1%	16.4%	18.4%	5.4	22

**Intermediate family history (affected FDR age 40)**	None	No MHT	**0.5%**	**1.0%**	**1.5%**	**2.1%**	**2.6%**	**5.4%**	**8.4%**	**11.5%**	**14.1%**	**16.4%**	6.1	—

	Oestrogen only	MHT used age 50.0–51.0	0.7%	1.2%	1.7%	2.3%	2.9%	5.8%	9.0%	12.3%	14.8%	17.1%	5.8	140
		MHT used age 50.0–55.0	0.6%	1.3%	1.9%	2.7%	3.3%	6.3%	9.5%	12.8%	15.4%	17.6%	5.7	80
												
		MHT used age 50.0–60.0	0.6%	1.3%	1.9%	2.7%	3.3%	6.7%	10.0%	13.4%	16.0%	18.2%	5.5	55

	Combined — all types	MHT used age 50.0–51.0	0.7%	1.1%	1.6%	2.2%	2.8%	5.7%	8.9%	12.1%	14.8%	17.1%	5.8	140
		MHT used age 50.0–55.0	0.8%	1.6%	2.4%	3.3%	4.1%	7.4%	10.8%	14.4%	16.9%	19.1%	5.2	37
		MHT used age 50.0–60.0	0.8%	1.6%	2.4%	3.3%	4.1%	9.5%	13.3%	17.3%	19.7%	21.9%	4.6	18

**Strong family history (two affected FDR age 50)**	None	No MHT	**0.7%**	**1.3%**	**2.0%**	**2.7%**	**3.4%**	**7.0%**	**10.6%**	**14.2%**	**17.1%**	**19.6%**	5.1	—

	Oestrogen only	MHT used age 50.0–51.0	0.9%	1.6%	2.3%	3.0%	3.8%	7.6%	11.4%	15.1%	18.0%	20.5%	4.9	114
		MHT used age 50.0–55.0	0.9%	1.7%	2.6%	3.4%	4.3%	8.2%	12.0%	15.8%	18.7%	21.1%	4.7	66
		MHT used age 50.0–60.0	0.9%	1.7%	2.6%	3.4%	4.3%	8.6%	12.6%	16.6%	19.4%	21.8%	4.6	45

	Combined — all types	MHT used age 50.0–51.0	0.9%	1.4%	2.1%	2.9%	3.6%	7.4%	11.2%	15.0%	18.0%	20.5%	4.9	114
		MHT used age 50.0–55.0	1.1%	2.1%	3.2%	4.3%	5.4%	9.5%	13.6%	17.7%	20.5%	22.9%	4.4	30
		MHT used age 50.0–60.0	1.1%	2.1%	3.2%	4.3%	5.4%	12.2%	16.8%	21.3%	23.9%	26.2%	3.8	15

*FDR = first-degree relative. MHT = menopausal hormone therapy. Cumulative risks are presented: the proportion of individuals expected to develop breast cancer from age 50.0 years to the current age specified. Family history parameters include the number of first-degree relatives affected by breast cancer (one or two) and their age at diagnosis (40, 50, or 60 years). MHT use parameters include type of MHT used and age of use.*

**Table 1b.** Cumulative risk of developing breast cancer from age 50.0–80.0 years, according to family history and MHT use for combined-continuous and combined-cyclical MHT

**Family** **history**	**MHT type**	**Current age, years**	**51.0**	**52.0**	**53.0**	**54.0**	**55.0**	**60.0**	**65.0**	**70.0**	**75.0**	**80.0**	**Likelihood of** **developing** **breast cancer** **age 50–80.** **One in:**	**Likelihood of** **developing breast** **cancer age 50–80** **attributable to** **MHT. One in:**
**Average woman**		Population risk	**0.3%**	**0.5%**	**0.8%**	**1.0%**	**1.3%**	**2.8%**	**4.4%**	**6.3%**	**8.1%**	**9.9%**	10.1	—

	None	No MHT	**0.2%**	**0.5%**	**0.7%**	**1.0%**	**1.2%**	**2.7%**	**4.3%**	**6.2%**	**8.0%**	**9.8%**	10.2	—

	Combined — continuous	MHT used age 50.0–51.0	0.3%	0.5%	0.7%	1.1%	1.3%	2.9%	4.6%	6.6%	8.5%	10.3%	9.8	219
		MHT used age 50.0–55.0	0.3%	0.8%	1.2%	1.7%	2.0%	3.8%	5.7%	8.0%	9.8%	11.5%	8.7	58
		MHT used age 50.0–60.0	0.3%	0.8%	1.2%	1.7%	2.0%	5.2%	7.4%	10.0%	11.7%	13.4%	7.5	28

	Combined — cyclical	MHT used age 50.0–51.0	0.3%	0.5%	0.7%	1.0%	1.3%	2.8%	4.5%	6.5%	8.3%	10.1%	9.9	307
		MHT used age 50.0–55.0	0.3%	0.7%	1.0%	1.5%	1.8%	3.5%	5.3%	7.5%	9.3%	11.0%	9.1	81
		MHT used age 50.0–60.0	0.3%	0.7%	1.0%	1.5%	1.8%	4.5%	6.5%	8.9%	10.7%	12.4%	8.1	38

**Modest family history** **(affected FDR age 60)**	None	No MHT	**0.4%**	**0.8%**	**1.2%**	**1.6%**	**2.0%**	**4.3%**	**6.7%**	**9.3%**	**11.7%**	**13.8%**	7.2	—

	Combined — continuous	MHT used age 50.0–51.0	0.5%	0.9%	1.3%	1.7%	2.1%	4.6%	7.2%	9.9%	12.4%	14.5%	6.9	146
		MHT used age 50.0–55.0	0.7%	1.4%	2.0%	2.7%	3.4%	6.1%	9.0%	12.0%	14.3%	16.4%	6.1	39
		MHT used age 50.0–60.0	0.7%	1.4%	2.0%	2.7%	3.4%	8.1%	11.4%	14.9%	17.1%	19.1%	5.2	19

	Combined — cyclical	MHT used age 50.0–51.0	0.5%	0.8%	1.3%	1.7%	2.1%	4.5%	7.0%	9.7%	12.2%	14.3%	7.0	204
		MHT used age 50.0–55.0	0.6%	1.2%	1.8%	2.4%	3.0%	5.6%	8.3%	11.2%	13.6%	15.7%	6.4	54
		MHT used age 50.0–60.0	0.6%	1.2%	1.8%	2.4%	3.0%	7.0%	10.1%	13.3%	15.6%	17.6%	5.7	26

**Intermediate family history (affected FDR age 40)**	None	No MHT	**0.5%**	**1.0%**	**1.5%**	**2.1%**	**2.6%**	**5.4%**	**8.4%**	**11.5%**	**14.1%**	**16.4%**	6.1	—

	Combined — continuous	MHT used age 50.0–51.0	0.7%	1.1%	1.6%	2.2%	2.8%	5.8%	9.0%	12.3%	15.0%	17.2%	5.8	120
		MHT used age 50.0–55.0	0.8%	1.7%	2.5%	3.5%	4.4%	7.7%	11.2%	14.8%	17.3%	19.6%	5.1	32
		MHT used age 50.0–60.0	0.8%	1.7%	2.5%	3.5%	4.4%	10.1%	14.1%	18.2%	20.6%	22.8%	4.4	16

	Combined — cyclical	MHT used age 50.0–51.0	0.6%	1.0%	1.6%	2.2%	2.7%	5.7%	8.8%	12.0%	14.7%	17.0%	5.9	168
		MHT used age 50.0–55.0	0.7%	1.5%	2.2%	3.1%	3.9%	7.0%	10.4%	13.9%	16.4%	18.7%	5.4	44
		MHT used age 50.0–60.0	0.7%	1.5%	2.2%	3.1%	3.9%	8.8%	12.5%	16.4%	18.8%	21.0%	4.8	22

**Strong family history (two affected FDR age 50)**	None	No MHT	**0.7%**	**1.3%**	**2.0%**	**2.7%**	**3.4%**	**7.0%**	**10.6%**	**14.2%**	**17.1%**	**19.6%**	5.1	—

	Combined — continuous	MHT used age 50.0–51.0	1.0%	1.4%	2.1%	2.9%	3.6%	7.5%	11.3%	15.1%	18.2%	20.6%	4.8	98
		MHT used age 50.0–55.0	1.2%	2.2%	3.4%	4.5%	5.7%	9.9%	14.1%	18.3%	21.1%	23.5%	4.3	26
		MHT used age 50.0–60.0	1.2%	2.2%	3.4%	4.5%	5.7%	13.0%	17.7%	22.4%	25.0%	27.3%	3.7	13

	Combined — cyclical	MHT used age 50.0–51.0	0.9%	1.4%	2.1%	2.8%	3.6%	7.3%	11.1%	14.9%	17.9%	20.3%	4.9	137
		MHT used age 50.0–55.0	1.0%	1.9%	3.0%	4.0%	5.1%	9.1%	13.1%	17.2%	20.0%	22.4%	4.5	36
		MHT used age 50.0–60.0	1.0%	1.9%	3.0%	4.0%	5.1%	11.3%	15.8%	20.1%	22.8%	25.2%	4.0	18

*FDR = first-degree relative. MHT = menopausal hormone therapy. Cumulative risks are presented: the proportion of individuals expected to develop breast cancer from age 50.0 years to the current age specified. Family history parameters include the number of first-degree relatives affected by breast cancer (one or two) and their age at diagnosis (40, 50, or 60 years). MHT use parameters include type of MHT used and age of use.*

**Table 2a.** Cumulative risk of death from breast cancer from age 50–80 years, according to family history and MHT use for oestrogen-only and combined-all MHT

**Family** **history of** **unaffected** **consultand**	**MHT use**		**For breast cancer diagnosed age 50–80 years**				

	**Type of** **MHT**	**Age of use**	**Cumulative risk** **of BC diagnosis**	**Total risk of breast** **cancer-specific death,** **%, likelihood**		**Absolute increase in risk of** **breast cancer-specific death** **due to MHT compared with** **no MHT, %, likelihood**	
**Average** **woman**	None	No MHT	9.8%	**1.7%**	1 in 58	—	—

	Oestrogen only	MHT used age 50.0–51.0	10.2%	**1.8%**	1 in 57	0.04%	1 in 2493
		MHT used age 50.0–55.0	10.5%	**1.8%**	1 in 56	0.07%	1 in 1406
		MHT used age 50.0–60.0	10.8%	**1.8%**	1 in 55	0.11%	1 in 940

	Combined — all types	MHT used age 50.0–51.0	10.2%	**1.8%**	1 in 57	0.04%	1 in 2376
		MHT used age 50.0–55.0	11.3%	**1.9%**	1 in 53	0.16%	1 in 642
		MHT used age 50.0–60.0	12.9%	**2.0%**	1 in 49	0.33%	1 in 305

**Modest family** **history** **(affected FDR** **age 60)**	None	No MHT	13.8%	**2.3%**	1 in 43	—	—

	Oestrogen only	MHT used age 50.0–51.0	14.4%	**2.4%**	1 in 42	0.06%	1 in 1648
		MHT used age 50.0–55.0	14.8%	**2.5%**	1 in 41	0.11%	1 in 936
		MHT used age 50.0–60.0	15.3%	**2.5%**	1 in 40	0.16%	1 in 635

	Combined — all types	MHT used age 50.0–51.0	14.4%	**2.4%**	1 in 41	0.06%	1 in 1551
		MHT used age 50.0–55.0	16.0%	**2.6%**	1 in 39	0.23%	1 in 429
		MHT used age 50.0–60.0	18.4%	**2.8%**	1 in 35	0.48%	1 in 208

**Intermediate** **family history** **(affected FDR** **age 40)**	None	No MHT	16.4%	**2.7%**	1 in 37	—	—

	Oestrogen only	MHT used age 50.0–51.0	17.1%	**2.8%**	1 in 36	0.07%	1 in 1361
		MHT used age 50.0–55.0	17.6%	**2.9%**	1 in 35	0.13%	1 in 761
		MHT used age 50.0–60.0	18.2%	**2.9%**	1 in 34	0.19%	1 in 522

	Combined — all types	MHT used age 50.0–51.0	17.1%	**2.8%**	1 in 36	0.08%	1 in 1281
		MHT used age 50.0–55.0	19.1%	**3.0%**	1 in 33	0.29%	1 in 350
		MHT used age 50.0–60.0	21.9%	**3.3%**	1 in 30	0.58%	1 in 173

**Strong family** **history** **(two affected** **FDR age 50)**	None	No MHT	19.6%	**3.2%**	1 in 31	—	—

	Oestrogen only	MHT used age 50.0–51.0	20.5%	**3.3%**	1 in 30	0.09%	1 in 1108
		MHT used age 50.0–55.0	21.1%	**3.4%**	1 in 30	0.16%	1 in 621
		MHT used age 50.0–60.0	21.8%	**3.4%**	1 in 29	0.23%	1 in 428

	Combined — all types	MHT used age 50.0–51.0	20.5%	**3.3%**	1 in 30	0.10%	1 in 1037
		MHT used age 50.0–55.0	22.9%	**3.6%**	1 in 28	0.35%	1 in 286
		MHT used age 50.0–60.0	26.2%	**3.9%**	1 in 26	0.70%	1 in 143

*FDR = first-degree relative. MHT = menopausal hormone therapy. Risks of breast cancer-specific death are presented: the proportion of individuals expected to die within 10 years from breast cancer diagnosed age 50.0–80.0 years. Family history parameters include the number of first-degree relatives affected by breast cancer (one or two) and their age at diagnosis (40, 50, or 60 years). MHT use parameters include type of MHT used and age of use.*

**Table 2b.** Cumulative risk of death from breast cancer from age 50–80 years, according to family history and MHT use for combined-continuous and combined-cyclical MHT

**Family** **history of** **unaffected** **consultand**	**MHT use**		**For breast cancer diagnosed age 50–80 years**				

	**Type of** **MHT**	**Age of use**	**Cumulative risk** **of BC diagnosis**	**Total risk of breast** **cancer specific death,** **%, likelihood**		**Absolute increase in risk of** **breast cancer specific death** **due to MHT compared to** **no MHT, %, likelihood**	
**Average** **woman**	None	No MHT	9.8%	1.7%	1 in 58		

	Combined – continuous	MHT used age 50.0–51.0	10.3%	1.8%	1 in 57	0.0%	1 in 2038
		MHT used age 50.0–55.0	11.5%	1.9%	1 in 53	0.2%	1 in 551
		MHT used age 50.0–60.0	13.4%	2.1%	1 in 48	0.4%	1 in 262

	Combined – cyclical	MHT used age 50.0–51.0	10.1%	1.8%	1 in 57	0.0%	1 in 2851
		MHT used age 50.0–55.0	11.0%	1.8%	1 in 54	0.1%	1 in 770
		MHT used age 50.0–60.0	12.4%	2.0%	1 in 50	0.3%	1 in 365

**Modest family** **history** **(affected FDR** **age 60)**	None	No MHT	13.8%	2.3%	1 in 43		

	Combined – continuous	MHT used age 50.0–51.0	14.5%	2.4%	1 in 41	0.1%	1 in 1331
		MHT used age 50.0–55.0	16.4%	2.6%	1 in 38	0.3%	1 in 369
		MHT used age 50.0–60.0	19.1%	2.9%	1 in 34	0.6%	1 in 179

	Combined – cyclical	MHT used age 50.0–51.0	14.3%	2.4%	1 in 42	0.1%	1 in 1861
		MHT used age 50.0–55.0	15.7%	2.5%	1 in 39	0.2%	1 in 514
		MHT used age 50.0–60.0	17.6%	2.7%	1 in 36	0.4%	1 in 249

**Intermediate** **family history** **(affected FDR** **age 40)**	None	No MHT	16.4%	2.7%	1 in 37		

	Combined – continuous	MHT used age 50.0–51.0	17.2%	2.8%	1 in 35	0.1%	1 in 1099
		MHT used age 50.0–55.0	19.6%	3.1%	1 in 33	0.3%	1 in 301
		MHT used age 50.0–60.0	22.8%	3.4%	1 in 29	0.7%	1 in 149

	Combined – cyclical	MHT used age 50.0–51.0	17.0%	2.8%	1 in 36	0.1%	1 in 1536
		MHT used age 50.0–55.0	18.7%	3.0%	1 in 34	0.2%	1 in 419
		MHT used age 50.0–60.0	21.0%	3.2%	1 in 31	0.5%	1 in 207

**Strong family** **history** **(two affected** **FDR age 50)**	None	No MHT	19.6%	3.2%	1 in 31		

	Combined – continuous	MHT used age 50.0–51.0	20.6%	3.3%	1 in 30	0.1%	1 in 890
		MHT used age 50.0–55.0	23.5%	3.6%	1 in 28	0.4%	1 in 246
		MHT used age 50.0–60.0	27.3%	4.0%	1 in 25	0.8%	1 in 123

	Combined – cyclical	MHT used age 50.0–51.0	20.3%	3.3%	1 in 30	0.1%	1 in 1243
		MHT used age 50.0–55.0	22.4%	3.5%	1 in 29	0.3%	1 in 343
		MHT used age 50.0–60.0	25.2%	3.8%	1 in 26	0.6%	1 in 170

*FDR = first-degree relative. MHT = menopausal hormone therapy. Risks of breast cancer-specific death are presented: the proportion of individuals expected to die within 10 years from breast cancer diagnosed age 50.0–80.0 years. Family history parameters include the number of first-degree relatives affected by breast cancer (one or two) and their age at diagnosis (40, 50, or 60 years). MHT use parameters include type of MHT used and age of use.*

For women with a family history of breast cancer, the baseline risk of breast cancer may be substantially increased. For example, for an unaffected 50-year-old consultand with a ‘strong’ family history (two first-degree relatives diagnosed at age 50 years), the cumulative breast cancer risk with no MHT is 7.0% to age 60, 14.2% to age 70, and 19.6% to age 80 years, increasing respectively to 9.1%, 17.2%, and 22.4% with 5 years (age 50–55 years) and to 11.3%, 20.1%, and 25.2% with 10 years (50–60 years) of combined-cyclical MHT. Therefore, 5/10 years of combined-cyclical MHT use confers an extra 1.3%/2.7% of absolute breast cancer risk to the woman of ‘average’ family history to age 70 years, but an extra 3.0%/5.9% to the woman with a ‘strong’ family history.

The baseline risk of dying from a breast cancer diagnosed at age 50–80 years is 1.7%/1.8%/2.0% (Table 2) for the woman of ‘average’ family history with no MHT/5 years MHT/10 years MHT (combined-cyclical MHT) respectively. For the woman with a ‘strong’ family history, these risks increase to 3.2%/3.5%/3.8%. Thus, for illustration, for 343 women with a ‘strong’ family history, approximately 11 would die from breast cancer diagnosed at age 50–80 years if none were taking MHT; if all 343 women took 5 years of combined-cyclical MHT then one additional woman of the 343 would die.

Compared with combined MHT, the relative risk of breast cancer is more modest for oestrogen-only MHT (see Supplementary Table S1 for details), meaning the estimates of cumulative absolute risk of breast cancer with administration of oestrogen-only MHT for those with a family history are also more modestly increased (Table 1). For a 50-year-old woman with a ‘strong’ family history, her breast cancer risk to age 70 years is increased from 14.2% with no MHT to 15.8%/16.6% with 5/10 years of oestrogen-only MHT, compared with 17.2%/20.1% with 5/10 years of combined-cyclical MHT. Cyclical versus continuous progestogen administration also makes a substantial difference (see Supplementary Table S1 for details) for the unaffected 50-year-old consultand with a ‘strong’ family history having 5 years of MHT at age 50–55 years, the risk of breast cancer by age 70 is estimated to be 17.2% with combined-cyclical MHT versus 18.3% with combined-continuous MHT, as compared to 14.2% with no MHT (Table 1).

## Discussion

### Summary

Patients with a significant (‘strong’) family history of breast cancer have a substantially increased baseline risk of developing the disease. However, most of the breast cancer incidence and mortality for this group will be attributable to their baseline risk rather than from the addition of MHT taken at age 50 years, even with a combined continuous preparation and even if taken for 10 years.

The impact of family history is greater at younger ages.^8^^,^^9^ This greater family history-related relative risk will therefore typically coincide with the greater relative risk for ‘current use’ of MHT, if administered at typical timing of menopause (about age 50 years). However, the baseline *absolute* risk of breast cancer is relatively lower during the 50–60 decade (particularly between the ages of 50 and 55 years) compared with age 60–80 years. Therefore, the increase in absolute risk of breast cancer is comparatively modest for 5 years of MHT administered at age 50–55 years, even for women with a ‘strong’ family history. The breast cancer risk also varies by preparation: risks are significantly lower for oestrogen-only MHT but the concomitant elevation in risk of endometrial cancer renders this option unsuitable except in women who have undergone hysterectomy.^1^ The risk is also reduced via cyclical rather than continuous administration of progestogens.^10^ Compared with the data presented for combined MHT, although not modelled here, the MHT-related increases in breast cancer risk would be anticipated to be attenuated for micronised progesterone and non-oral preparations.

Furthermore, breast cancer is typically associated with a comparatively good prognosis, especially for hormone-receptor positive disease, the subtype associated with MHT administration.^12^ Many people have limited understanding of the variability of disease-specific fatality for different cancer types: it may thus be of value to communicate the likelihood of dying from breast cancer as distinct from the likelihood of developing the disease. According to the model in this study, for a woman of ‘average’ family history and a woman with the ‘strong’ family history, administration of 5 years of combined-cyclical MHT will respectively increase their absolute risk of dying from a breast cancer (diagnosed at 50– 80 years) by 0.1% and 0.3% compared with no MHT.

Symptoms of menopause can be highly disabling: the near-term mitigation may be of high value compared against hypothetical possibility of future disease, even for a woman with a significant (‘strong’) family history. The illustrations of cumulative risk of breast cancer and concomitant impact on breast cancer-specific mortality for different patterns of MHT exposure and family history in this study will be informative for medical practitioners and patients in joint decision making regarding MHT prescription.

### Strengths and limitations

For the impact of MHT use on breast cancer risk, estimates were used for relative risks of breast cancer calculated from the CGHFBC study, which was a collaborative analysis of 24 prospective studies of MHT use involving 108 647 cases of female breast cancer, as this represented the largest and most detailed analysis identified.^10^ Notably, other analyses of MHT and breast cancer have reported broadly similar associations with varying magnitudes of effect sizes while other studies give different results.

A number of assumptions were required for modelling the risks for the different patterns of MHT administration (see Supplementary Table S2 for details). These include assumptions that the estimates of breast cancer relative risk derived from the CGHFBC were constant across a delineated period of ‘current use’ of MHT; were constant across a subsequent period of ‘past use’ to age 70 years; and that ‘legacy risk’ stopped at age 70 years. The MHT-associated risks were derived from data comprising a range of MHT preparations; subgroup analysis has enabled generation of metrics for two major groups (combined MHT and oestrogen-only), along with estimation of differences within combined preparations for cyclical versus continuous progestogen administration. Lower risks have been reported for more specific preparations, for example, those containing dydrogesterone and micronised progestogens (body-identical or non-synthetic), but insufficient resolution is available to allow analyses by different durations of exposure, and for current versus past risk.^10^^,^^13^ Furthermore, very few women in the CGHFBC were taking these preparations. Data were not available by which to evaluate non-oral MHT preparations, for example, transdermal oestrogens or progestogen-releasing hormonal intrauterine devices. In participants included in the CGHFBC study, <1% reported co-use of progestogen-releasing intrauterine device during the study or preceding 5 years, suggesting cross-contamination of these data for reported oestrogen-only MHT use is likely to be limited. The assumed baseline breast cancer risks for different family histories were based on modelling of familial breast cancer using segregation analysis methodologies and thus are not directly measured. However, the BOADICEA model has been extensively validated in independent prospective studies for predicting breast cancer risk on the basis of cancer family history, and is recommended for this purpose by NICE.^7^^,^^14^^,^^15^ The current study assumed that the effect of MHT and family history act multiplicatively on risk, which fits the retrospective risk modelling of MHT in the validation both for the BOADICEA model (as used in this study), and also for the Tyrer–Cuzick model (another breast cancer risk prediction model). Notably, the only interaction reported in the CGHFBC was that between adiposity and risk of oestrogen-only MHT.^10^ It has also been assumed for excess breast cancers arising due to MHT that the mortality rates are those for ER-positive cancers, for which survival is better than for other lower-frequency breast cancer subtypes.^12^

More extensive patterns of family history were not investigated and the effects of other breast cancer risk factors, such as breast density, body mass index (BMI), alcohol consumption, and physiological endocrinological factors (such as age of menarche, number of pregnancies, and duration of lactation), were not considered. Therefore, the estimates presented would be applicable to an average woman in the population with respect to these variables. It was not possible to focus on subgroups delineated by ethnicity: by which baseline breast cancer risk, breast cancer mortality, and MHT use are reported to vary.^16^ Furthermore, the impact of carrying pathogenic variants in breast cancer susceptibility genes such as BRCA1 and BRCA2 was not considered, but such women would typically be managed in clinical genetics clinics. Analyses were restricted to a limited number of scenarios of MHT administration with regard to age of initiation and duration of exposure. Risks are presented up to age 80 years because of the proximity to median life expectancy (nevertheless, approximately 20% of all breast cancers are diagnosed beyond the age of 80 years).^11^ The current study also focuses exclusively on the MHT-associated risk of breast cancer as this is related to breast cancer family history but does not consider other health risks or benefits associated with MHT, for example those relating to cardiovascular disease, thrombo-embolism, or ovarian cancer.

### Comparison with existing literature

For comprehensive individual breast cancer risk estimation, incorporation of the specific individual details of family history, genetic testing, breast density, BMI, and other factors is required, for which the IBIS (Tyrer Cuzick) tool allows incorporation of both past and proposed future MHT use, while the CanRisk (BOADICEA) interactive tool considers past and current MHT use only.^8^^,^^9^^,^^17^^–^19 However, these are dynamic tools, designed for interactive individual-patient level use. None currently allows for the range of MHT formulations and durations of use considered here. These tools focus only on incidence and do not consider breast cancer-specific mortality.

### Implications for practice

The illustrations of cumulative risk of breast cancer and concomitant impact on breast cancer-specific mortality for different patterns of MHT exposure and family history in this study will be informative for medical practitioners and patients in joint decision making regarding MHT prescription.

It is potentially challenging for patients to interpret complex data about risk. A relative risk may sound substantial, but the change in absolute risk may be modest if the baseline risk is low. A patient’s perception of risk will potentially be influenced by individual, cultural, and experiential factors, and is inevitably subjective and context dependent. Some patients may be interested in the shorter-term disease risk over the next 5 or 10 years. Other patients may wish to contextualise this risk in terms of risk over a lifetime (or at least up to age 80 years). Some women with a family history of breast cancer may see the additional MHT-related risk as modest in comparison to the baseline risk. Others may seek to avoid any further increase in risk from modifiable factors, especially if they are at a very high baseline risk because of their family history. The data in this study illustrate the comparatively modest risks of breast cancer incidence and mortality associated with a single year of MHT administration, even for those with a ‘strong’ family history. These data may be reassuring for women experiencing severe menopausal symptoms who may wish to first explore the extent of symptom mitigation that is achievable.

In future, patients and clinicians may benefit from higher-resolution data covering different preparations of oestrogen and progestogen, in particular, non-systemic routes.
